# Annotations

**Published:** 1893-08-19

**Authors:** 


### ANNOTATIONS.

**Practical Education.**

Mr. Craik, C.B., the Secretary of the Scotch Education Department, has drawn up a code of regulations for evening schools, which should please at once the most advanced Radical and the most severe Conservative. Evening schools were in the beginning meant to give an opportunity of acquiring the three R's to those who had had no chance of learning in their youth; but now that the most industrious ignorance cannot wholly evade the acquisition of knowledge, the question arises if their utility cannot be extended. The question is answered in this code. Strictly intellectual work is by no means omitted from it; it is still to be regarded as a means of supplying defects in the elementary education of certain scholars; but it is intended as a means of enabling scholars who have left elementary schools to acquire something more than elementary knowledge, and especially of giving them opportunities of learning the scientific principles which underlie the employment upon which they have entered. In short, the evening school is to be as far as possible a technical school, with special adaptation to local industries. In an agricultural district the boys will be taught horticulture and the girls dairy work. In seaside towns instruction will be given in navigation; clerks will learn book-keeping and shorthand, and many industries will be served by the teaching of chemistry, electricity, and mechanics. We have spoken of the pupils as boys and girls, but as a matter of fact there is no restriction as to age, the old rule which confined the Government grant to pupils under twenty-one having been rescinded in face of a development by which it is likely that those who have grown to manhood and womanhood are most likely to profit. Certain subjects are to be taught to one sex only. In spite of all that is said of the actual or imminent reversal of the positions of the sexes, men are not to be taught domestic economy nor women agriculture; but since the latter are certainly beginning to take a more active part in public life, it is good to see that both sexes are to be instructed in "the life and duties of a citizen." If this code in any way succeeds in what it aims at—and so thoughtfully is it drawn up that we cannot doubt its success—it should produce good citizens; men and women who understand in principle and practice the work they are engaged in, and who appreciate their privileges and duties in relation to the county to which they belong. A code like this is worth a great deal of obstreperous legislation in promoting the well-being of a community.

**Laundry-Work for Feeble-Minded Girls.**

We have to congratulate the people of Birmingham on the establishment of the Laundry and Homes of Industry at Arrowfield Top. These homes have been opened only a year, and now hold their full complement of ten girls, but there have been 119 applications for admission. The inmates are girls of a type which we dealt with in The Hospital a few weeks ago, those feeble-minded creatures, not idiots, but just so far short of the average standard of intelligence, as to be incapable of guiding themselves through life. Taken by the hand, protected, controlled, they can be taught to earn their living, at least in part; they are not mere burdens on the community. Left to themselves, they are worse than burdens; they sink into pauperism after having transmitted their feeble brains and deficient moral natures to their often nameless children. How large a proportion of our pauper population such women form, is hardly realized. Miss Clifford, a Guardian at Barton Regis, stated at the first meeting of the Arrowfield Top Homes, that she had found on investigation that a third of the able-bodied women in the workhouse belonged to this class; and it is not to be thought that Barton Regis is in this matter unique. These women are not naturally degraded; they are, on the contrary, kindly and affectionate, and too confiding for independent life. But the experiment has been made and proved in America and on the Continent, that in affectionate dependence they are happy, willing, and, in their degree, serviceable. England, charitable, philanthropic England, has been late in seeing its duty to the feeble-minded girls; but the success of Arrowfield Top shows not only the value of the work, but the great and pressing need for it. There are many things that can be done without great intelligence. The rougher kinds of laundry work, plain knitting, simple basket making, almost any industry that can be taught to children may be acquired by those who are, literally, "children of a larger growth." Their intelligence will develop with their work. A certain amount of intellectual training may also benefit them; at least they will profit by the habit of attention and self-control, and by association with others under, of course, efficient and kindly supervision. But in all probability their fingers will always be nimbler than their brains. The great point is to get hold of them when they are young, before they have been degraded by contact with an evil and pitiless world. This is what is attempted at Arrowfield Top. Alas! that there should be but one Arrowfield Top, with accommodation for only ten girls, when twelve times that number are knocking at the door.

**Overcrowding in Lanarkshire.**

We have more than once drawn attention to the conditions of working-class life in the Scottish "black country," and for our pains have been assured that we are misrepresenting an earthly paradise. But, to use a quotation than which none is more popular across the border, "facts are chiels that winna ding," and the facts given in the following extract from a recent number of the *Glasgow Herald* are enough to startle the average believer in letting well alone: "Coatbridge. — Overcrowding with Lodgers. — Margaret Lithgow or Sword was fined 10s., or seven days, for keeping five lodgers in her one apartment house in addition to herself and three children, being nine persons in space for three and a half.—James Carr, miner, General's Row, for keeping six lodgers in his one room house, besides his own family (eleven persons in a house size for four), was fined 15s., or ten days.—James Malone, miner, General's Row, and Isaac Hutton, miner, were each fined 10s., or seven days, for similarly overcrowding their houses." Sensitive people had better not try to picture to themselves the moral and physical condition of the inhabitants of these domiciles. The truth is that the well-to-do classes in these districts are nearly as indifferent to this shameful overcrowding as the offenders themselves. The miner's tenure of his house is so uncertain that he has for it little of the feeling of home. If he goes on strike he is liable to be turned out of doors, even if he is able and willing to pay rent; his house is part of his wage, and if he loses his work he must lose it too. The fate of widows in these communities is particularly hard. If a miner dies, his house is immediately wanted for someone else, and his widow may be turned out at a fortnight's notice. Picture an ignorant woman, probably with several young children, turned out to the street. Is it strange that if she can keep possession of her house by taking one or two of the unmarried miners as lodgers, thus making it of use to the owner, she should be tempted to do so? The practice begun for convenience is continued for profit, and the result is such shameful overcrowding as we have recorded, with unspeakable evils in its train. We regret that the law does not punish it more severely, put it is something—and something new—for it to be punished at all.

